# Polyphenolic Composition and Evaluation of Antioxidant Activity, Osmotic Fragility and Cytotoxic Effects of *Raphiodon echinus* (Nees & Mart.) Schauer

**DOI:** 10.3390/molecules21010002

**Published:** 2015-12-29

**Authors:** Antonia Eliene Duarte, Emily Pansera Waczuk, Katiane Roversi, Maria Arlene Pessoa da Silva, Luiz Marivando Barros, Francisco Assis Bezerra da Cunha, Irwin Rose Alencar de Menezes, José Galberto Martins da Costa, Aline Augusti Boligon, Adedayo Oluwaseun Ademiluyi, Jean Paul Kamdem, João Batista Teixeira Rocha, Marilise Escobar Burger

**Affiliations:** 1 Centro de Ciências Biológicas e da Saúde-CCBS, Departamento de Ciências Biológicas, Universidade Regional do Cariri (URCA), Pimenta, Crato CEP 63.100-000, CE, Brazil; duarte105@yahoo.com.br (A.E.D.); lmarivando@hotmail.com (L.M.B.); cunha.urca@gmail.com (F.A.B.C.); 2 Programa de Pós-Graduação em Bioquímica Toxicológica, Departamento de Bioquímica e Biologia Molecular, Universidade Federal de Santa Maria, Santa Maria 97105-900, RS, Brazil; memypw@yahoo.com.br (E.P.W.); aoademiluyi@futa.edu.ng (A.O.A.); kamdemjeanpaul2005@yahoo.fr (J.P.K.); 3 Departamento de Fisiologia e Farmacologia, Universidade Federal de Santa Maria, Santa Maria 97105-900, RS, Brazil; katianeroversi@gmail.com; 4 Laboratório de Botânica Aplicada, Departamento de Ciências Biológicas, Universidade Regional do Cariri (URCA), Pimenta, Crato CEP 63.100-000, CE, Brazil; arlene.pessoa@urca.br; 5 Laboratório de Farmacologia e Química Molecular, Departamento de Química Biológica, Universidade Regional do Cariri, Pimenta, Crato CEP 63.100-000, CE, Brazil; irwin.alencar@urca.br; 6 Laboratório de Pesquisas de Produtos Naturais, Departamento de Química Biológica, Universidade Regional do Cariri, Crato CEP 63.105.000, CE, Brazil; galberto.martins@urca.br; 7 Laboratório de Fitoquímica, Departamento de Farmácia Industrial, Universidade Federal de Santa Maria, Santa Maria 97105-900, RS, Brazil; alineboligon@yahoo.com.br; 8 Functional Foods and Nutraceutical Unit, Department of Biochemistry, Federal University of Technology, P.M.B. 704, Akure 340001, Nigeria; 9 Departamento de Bioquímica, Instituto de Ciências Básica da Saúde, Universidade Federal do Rio Grande do Sul, Porto Alegre CEP 90035-003, RS, Brazil; jpkamdem@gmail.com

**Keywords:** *Raphiodon echinus*, antioxidant activity, phenolic acids, HPLC-DAD

## Abstract

*Raphiodon echinus* (*R. echinus*) is used in Brazilian folk medicine for the treatment of inflammation, coughs, and infectious diseases. However, no information is available on the potential antioxidant, cytotoxicity and genotoxicity of this plant. In this study, the polyphenolic constituents, antioxidant capacity and potential toxic effects of aqueous and ethanolic extracts of *R. echinus* on human erythrocytes and leukocytes were investigated for the first time. *R. echinus* extracts showed the presence of Gallic, chlorogenic, caffeic and ellagic acids, rutin, quercitrin and quercetin. Aqueous and ethanolic extracts of *R. echinus* exhibited antioxidant activity in DPPH radical scavenging with IC_50_ = 111.9 μg/mL (EtOH extract) and IC_50_ = 227.9 μg/mL (aqueous extract). The extracts inhibited Fe^2+^ (10 μM) induced thiobarbituric acid reactive substances (TBARS) formation in rat brain and liver homogenates. The extracts (30–480 μg/mL) did not induce genotoxicity, cytotoxicity or osmotic fragility in human blood cells. The findings of this present study therefore suggest that the therapeutic effect of *R. echinus* may be, in part, related to its antioxidant potential. Nevertheless, further *in vitro* and *in vivo* studies are required to ascertain the safety margin of its use in folk medicine.

## 1. Introduction

Some medicinal plants used in folk medicine can cause toxicity to humans and also exhibit carcinogenicity and genotoxicity [1,2]. Therefore, toxicological studies of plant extracts used in traditional medicine are highly recommended, as it is part of the procedures that contribute to the standardization of phytopharmaceuticals [3,4].

The genus *Raphiodon* (*Lamiaceae*) is represented by only one species, *Raphiodon echinus* (*R. echinus*)*,* which is common to Eastern Brazil and typical of the “caatinga” (semi-arid vegetation) [5]. It is a prostrate herb with aromatic leaves and long pedunculate spherical heads with bright purple flowers, found in the states of Bahia, Pernambuco, Paraíba, Ceará and Minas Gerais. The infusion of the leaves of *R. echinus* is used in Brazilian folk medicine for the treatment of inflammation, coughs and infectious diseases. Studies have shown that *R. echinus* exhibits antimicrobial [6], anti-inflammatory and analgesic activities [7]. These biological properties are generally attributed (at least in part) to the antioxidant activity. However, to the best of our knowledge, there are no reports on the antioxidant activity of this plant extract.

Free radicals are thought to be important mediators of tissues injury under pathological conditions [8]. Byproducts of lipid peroxidation (LPO) have been shown to decrease cell membrane fluidity, inactivation of membrane-bound enzymes and loss of essential fatty acids [9], resulting in increased osmotic fragility of the cell [10] Consequently, the use of plant extracts or compounds that can act as “physical barriers” to prevent free radicals generation from important sites (e.g., cell membranes), or able to inhibit the propagation of LPO are of utmost importance.

Given that there is limited literature information regarding the biological activities of *R. echinus*, especially its antioxidant activity, and no information on its potential toxic effects to human blood cells, the present study, therefore, aimed at investigating for the first time the antioxidant capacity, iron chelating activity, cytotoxicity, and genotoxicity of *R. echinus* leaf extracts (aqueous and ethanolic) in human leukocytes, as well as its effect on osmotic fragility in human erythrocytes. Furthermore, polyphenolic constituents that may be at least, in part, responsible for the beneficial effects of *R. echinus* extracts were characterized using high performance liquid chromatography coupled to diode array detector (HPLC-DAD). This study is particularly important in view of the fact that it provides supportive information on the use of this plant in traditional medicine.

## 2. Results and Discussion

The lack of scientific evidence for the biological activities and safety profile of plant extracts used in traditional medicine have generated considerable concern in the scientific community. In fact, it is imperative to isolate those plants that can represent serious public health problem. In the present study, we investigated for the first time the potential antioxidant activity of *R. echinus* leaves extracts as well as its potential cytotoxic and genotoxic effects in human leukocytes. In addition, the influence of *R. echinus* on human erythrocytes and the polyphenolic constituents of the leaves extracts were characterized and reported for the first time.

### 2.1. HPLC Characterization of the Polyphenolic Constituents of Aqueous and Ethanolic Extracts of R. echinus Leaves

**Figure 1:**
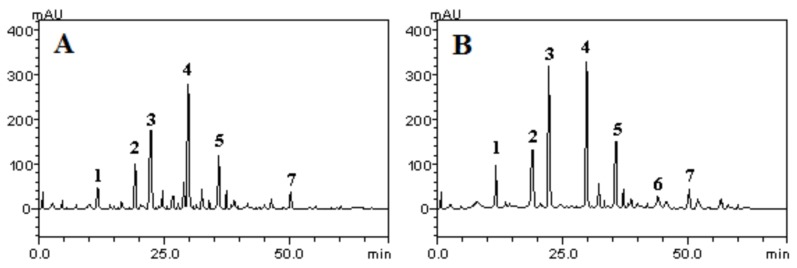
HPLC-DAD chromatograms of aqueous (**A**) and ethanolic (**B**) extracts of the leaves of *Raphiodon echinus* (*R. echinus*)*:* Gallic acid (peak 1), chlorogenic acid (peak 2), caffeic acid (peak 3), ellagic acid (peak 4), rutin (peak 5), quercitrin (peak 6) and quercetin (peak 7). Calibration curve for Gallic acid: *y* = 12574*x* + 1307.8 (*r* = 0.9999); chlorogenic acid: *y* = 11953*x* + 1278.2 (*r* = 0.9995); caffeic acid: *y* *=* 11976*x* + 1187.0 (*r* = 0.9996); ellagic acid: *y* = 13169*x* + 1346.8 (*r* = 0.9999); quercitrin: *y* = 12473*x* + 1187.5 (*r* = 0.9991); rutin: *y* = 12814*x* + 1189.3 (*r* = 0.9999) and quercetin: *y* = 12537*x* + 1375.6 (*r* = 0.9994). All chromatography operations were carried out at ambient temperature and in triplicate.

The HPLC profile of the aqueous and ethanolic extracts of the leaves of *R. echinus* revealed the presence of polyphenolic constituents, which appeared with the following elution profile/order: Gallic acid (Rt = 7.12 min, peak 1), chlorogenic acid (Rt = 19.34 min, peak 2), caffeic acid (Rt = 22.61 min, peak 3), ellagic acid (Rt = 29.73 min, peak 4), rutin (Rt = 36.12 min, peak 5), quercitrin (Rt = 43.95 min, peak 6) and quercetin (Rt = 50.03 min, peak 7) (Figure 1A,B). However, quercitrin appeared to be absent in the aqueous extract (Figure 1A, Table 1) and present in the ethanolic extract of *R. echinus* (Figure 1B, Table 1). The identification of these constituents was made by comparing their retention time and UV spectra of the peaks in the samples with those of authentic reference samples or isolated compounds. Based on our results, ellagic acid appeared to be the major component of both extracts with 79.13 and 63.18 mg/g in ethanolic and aqueous extract, respectively. In contrast, quercetin (9.87 mg/g; representing about 0.98% of the aqueous extract) and quercitrin (7.12 mg/g; representing 0.81% of the ethanolic extract) were the two components detected in the smallest quantities in *R. echinus* extracts (Table 2).

**Table 1:** Schedule of evaluation of oxidation or chelation of Fe^2+^/Fe^3+^ by plant extracts.

Time	Sequence of Addition	Reading at 510 nm
0 min	Extract (30–120 μg/mL)	-
	FeSO_4_ (110 μM)	
10 min	*Ortho*-phenanthroline (0.25%)	-
10 min	Immediately after mixing with	First reading (0 min)
	*Ortho*-phenanthroline	
20 min	-	Second reading (10 min)
30 min	-	Third reading (20 min)
30 min	Ascorbic acid (AA, final concentration, 5 mM)	-
35 min	-	First reading after AA (25 min)
40 min	-	Second reading after AA (30 min)
50 min	-	Third reading after AA (40 min)

**Table 2:** Phenolics and flavonoids composition of *R. echinus* extracts.

Compounds	Aqueous Extract/mg·g^−1^ (%)	Ethanolic Extract/mg·g^−1^ (%)	LOD/μg·mL^−1^	LOQ/μg·mL^−1^
Gallic acid	11.59 ± 0.01 ^a^ (1.15)	25.03 ± 0.01 ^a^ (2.50)	0.011	0.037
Chlorogenic acid	25.07 ± 0.02 ^b^ (2.50)	31.94 ± 0.02 ^b^ (3.19)	0.009	0.035
Caffeic acid	40.19 ± 0.02 ^c^ (4.01)	76.45 ± 0.02 ^c^ (7.64)	0.026	0.090
Ellagic acid	63.18 ± 0.01 ^d^ (6.31)	79.13 ± 0.03 ^c^ (7.91)	0.017	0.056
Rutin	26.50 ± 0.03 ^b^ (2.65)	35.84 ± 0.02 ^b^ (3.58)	0.024	0.080
Quercitrin	-	7.12 ± 0.01 ^d^ (0.81)	0.035	0.118
Quercetin	9.87 ± 0.03 ^a^ (0.98)	12.37 ± 0.01 ^e^ (1.23)	0.019	0.063

Results are expressed as mean ± standard deviations (SD) of three determinations. Different letters in the same column indicate significant difference by Tukey test at *p* < 0.01. LOD: limit of detection, LOQ: limit of quantification.

### 2.2. Antioxidant Activity

#### 2.2.1. Scavenging Effect of *R. echinus* Extracts on DPPH Radical

**Figure 2:**
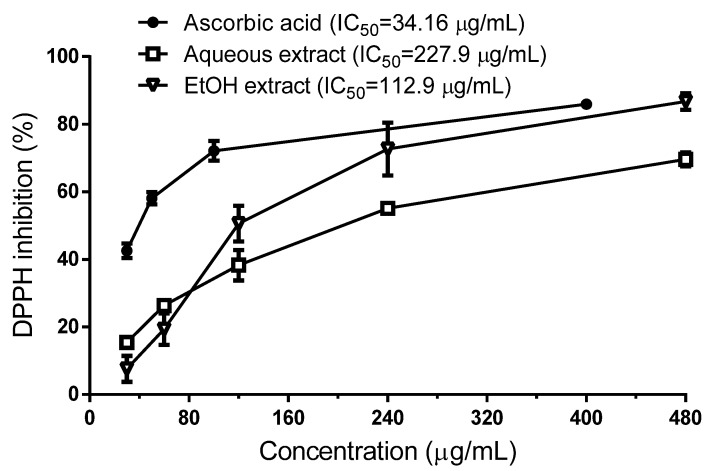
Quenching of DPPH radicals by aqueous and ethanolic extracts from the leaves of *R. echinus*. Data are expressed as mean ± SEM of *n* = 4 independent experiments.

Aqueous and ethanolic (EtOH) extracts of *R. echinus* exhibited antioxidant activity against DPPH radical in a concentration-dependent manner (Figure 2). According to the calculated IC_50_ values, the ethanolic extract exhibited stronger DPPH radical scavenging activity than aqueous extract, which was about two times higher than that of aqueous extract (Figure 2).

In this assay, the bleaching of the DPPH coloration is an indication of the free radical scavenging capacity of the samples. Our results revealed that the ethanolic extract showed greater antioxidant activity than aqueous extract (IC_50_ = 112.9 μg/mL *vs.* 227.9 μg/mL). However, this activity was three times lower than that of ascorbic acid (IC_50_ = 34.16 μg/mL), used as a standard. The higher antioxidant capacity of the ethanolic extract compared to the aqueous extract can be explained by its higher polyphenolic contents. In line with this, the total phenolic content of both extracts assayed by the Folin–Ciocalteu method revealed that ethanolic extract exhibit higher total phenolic content (TPC) than aqueous extract (Table 3). Numerous studies have described a positive correlation between the antioxidant activity and phenolic content [11,12]. Although we did not perform such correlation calculations, the results obtained here clearly demonstrate that ethanolic extract, which exhibited high TPC, has a stronger antioxidant activity by DPPH radical scavenging.

**Table 3:** Total phenolic content of *R. echinus* extracts.

	*R. echinus*	
	Aqueous Extract	Ethanolic Extract
Total phenolics (mg GAE/g dry extract)	173.0 ± 0.07	389.1 ± 0.04

Results are expressed in milligram Gallic acid equivalent (GAE) per gram of dry extract; *n* = 3.

#### 2.2.2. Effect of *R. echinus* Extracts on Fe^2+^ Induced Lipid Peroxidation (LPO) in the Rat Brain and Liver Homogenates

**Figure 3:**
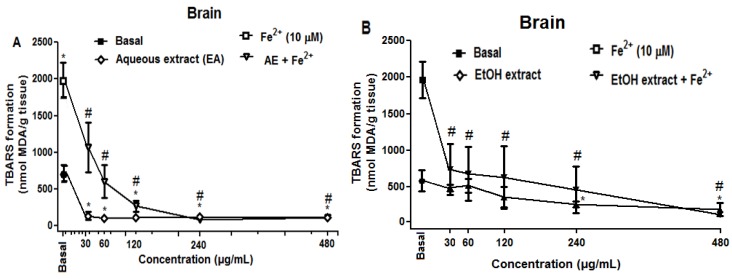
Effect of aqueous (**A**) and ethanolic (**B**) extracts of the leaves of *R. echinus* on lipid peroxidation induced by iron in rat brain homogenates. The homogenate was incubated for 1 h with Fe^2+^ (10 μM) in the presence or absence of different concentrations of the extracts. Values represent the mean of *n* = 3 independent experiments performed in duplicate ± SEM. *: *p* < 0.05 *vs.* basal and #: *p* < 0.05 *vs.* Fe^2+^.

Fe^2+^ induced a significant increase in TBARS production in brain homogenates (*p* < 0.05; Figure 3). However, the aqueous (Figure 3A) and ethanolic (Figure 3B) extracts of *R. echinus* significantly reduced the LPO in concentration-dependent manner both under basal and iron-stimulated conditions (Figure 3A,B).

**Figure 4:**
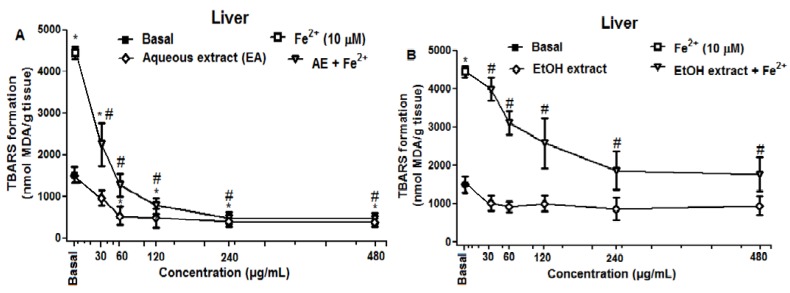
Effect of aqueous (**A**) and ethanolic (**B**) extracts of the leaves of *R. echinus* on lipid peroxidation induced by iron in rat liver homogenates. The homogenate was incubated for 1 h with Fe^2+^ (10 μM) in the presence or absence of different concentrations of the extracts. Values represent the mean of *n* = 3 independent experiments performed in duplicate ± SEM. *: *p* < 0.05 *vs.* basal and #: *p* < 0.05 *vs.* Fe^2+^.

Similar to that observed with brain homogenates, crude extracts of *R. echinus* inhibited LPO induced by Fe^2+^ in rat liver homogenates (Figure 4). In contrast, ethanolic extract (EtOH extract) did not inhibited TBARS formation under basal condition (Figure 4B), while aqueous extract (AE) inhibited TBARS production under basal conditions (Figure 4A).

The results demonstrated that aqueous and ethanolic extracts from the leaves of *R. echinus* exhibited protective effects against Fe^2+^ induced lipid peroxidation (LPO) in rat brain homogenates. Although iron (II) is an essential element for life, free Fe^2+^ in biological systems, however, can be toxic [13] and its levels have been shown to be increased in many neurological disorders including Alzheimer’s and Parkinson’s diseases [14,15,16]. The results of the current study demonstrated that both extracts showed antioxidant activity against Fe^2+^ induced LPO in rat brain and liver homogenates at all the concentrations tested. Nevertheless, the ability of these extracts to inhibit LPO could be attributed (at least in part) to the capacity of their chemical constituents to chelate/inactivate Fe^2+^, thereby, preventing or reducing reactive oxygen species generation. The HPLC fingerprint of these extracts revealed the presence of phenolic acids (Gallic, ellagic, chlorogenic and caffeic acids) and flavonoids (quercetin, quercitrin and rutin), compounds that are known scavengers and inhibitors of LPO [17,18]. Of particular importance, is the finding which revealed that chlorogenic and caffeic acids could inhibit free radicals formation through several mechanisms including the reaction complex formation with iron ions in the reactions of hydrogen peroxide with iron (II), hydrogen peroxide with ferric iron and 3-hydroxyanthranilic acid [19]. In both tissues, aqueous extract showed highest antioxidant activity against Fe^2+^ induced LPO by significantly reducing TBARS formation at much lower concentrations when compared to the ethanolic extract. At basal conditions, ethanolic extract did not have any effect on TBARS formation in the liver, while it did in the brain at the higher concentrations tested (240 and 480 μg/mL).

#### 2.2.3. Iron Chelating Potential of *R. echinus* Extracts

**Figure 5:**
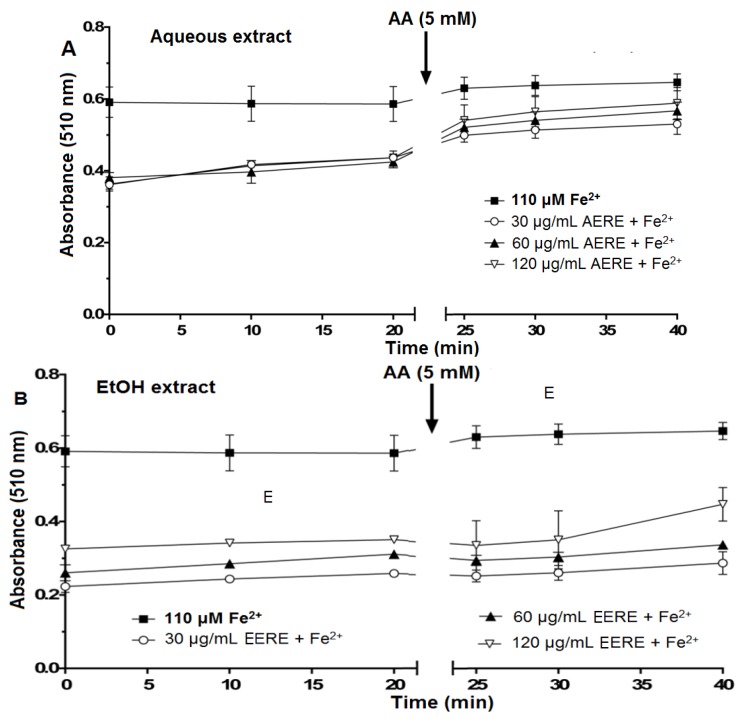
Oxidation of Fe^2+^ by aqueous (**A**) and ethanolic (**B**) extracts from the leaves of *R. echinus* (1–60 μg/mL). The extracts (30–120 μg/mL) were incubated with FeSO_4_ (110 μM) for 10 min. Then, *ortho*-phenanthroline was added and the absorbance of the reaction mixture was measured at 0, 10 and 20 min following its addition. After the last reading (at 20 min), 5 mM ascorbic acid (AA) was added to the reaction mixture, and the absorbance was read again after 5 min (at 25 min), 10 min (at 30 min) and 20 min (at 40 min) (see Table 1 for details). Values represent the mean ± SEM of three independent experiments performed in duplicate. AERE, aqueous extract of *R. echinus*; EERE, ethanolic extract of *R. echinus*.

The incubation of aqueous and ethanolic extracts with Fe^2+^ caused a decreased in the absorbance at 510 nm with the effect most apparent from 30 to 120 μg/mL (Figure 5A,B). The addition of ascorbic acid caused only a modest increase in the absorbance at 510 nm and this was a little more apparent for the aqueous extract (Figure 5A). The most plausible interpretation of these results is that the extracts can chelate Fe^2+^ and accelerate the oxidation of Fe^2+^ to Fe^3+^. However, this Fe^3+^ was only partially or not released from the complex, as otherwise ascorbic acid should have reduced Fe^3+^ to Fe^2+^, resulting in the formation of the complex between Fe^2+^ and *ortho*-phenanthroline. However, this occurred only to a limited extent.

To verify whether the decrease in the absorbance in the presence of aqueous (Figure 5A) or ethanolic (Figure 5B) extracts was caused by the chelation or oxidation of Fe^2+^, ascorbic acid (AA) was added to the reaction medium after 20 min of incubation of Fe^2+^ with the extracts and *ortho*-phenanthroline. The objective was to reduce Fe^3+^ that could have being formed during the reaction back to Fe^2+^. In the presence of aqueous extract (Figure 5A) or ethanolic extract (Figure 5B), the addition of AA to the reaction mixture caused a partial increase in the absorbance after 5, 10 and 20 min of incubation, which is an indication of stimulated oxidation of Fe^2+^ by the extracts.

Earlier reports have shown the ability of antioxidants to chelate or deactivate transition metals, thus preventing them from initiating lipid peroxidation and oxidative stress via metal-catalyzed reaction [20]. Chelation of transition metals such as iron can be viewed as a preventive antioxidant mechanism. In the present study, both extracts chelate Fe^2+^ partially and possibly converted it to Fe^3+^. However, Fe^3+^ was only partially released from the plant extract-iron complex as evidenced by the small increase in the absorbance after addition of the reducing agent, ascorbic acid (AA) to the reaction medium. Furthermore, if there was an appreciable free Fe^3+^ pool, this should have been reduced back to Fe^2+^ in the presence of ascorbic acid. However, this was very modest. Consequently, it is possible to presume that the observed inhibition of Fe^2+^ induced LPO in the presence of the extracts was a result of both direct interaction with free radicals and via chelation of Fe^2+^/Fe^3+^ species.

### 2.3. Cytotoxicity Effect of R. echinus Extracts on Human Leukocytes

**Figure 6:**
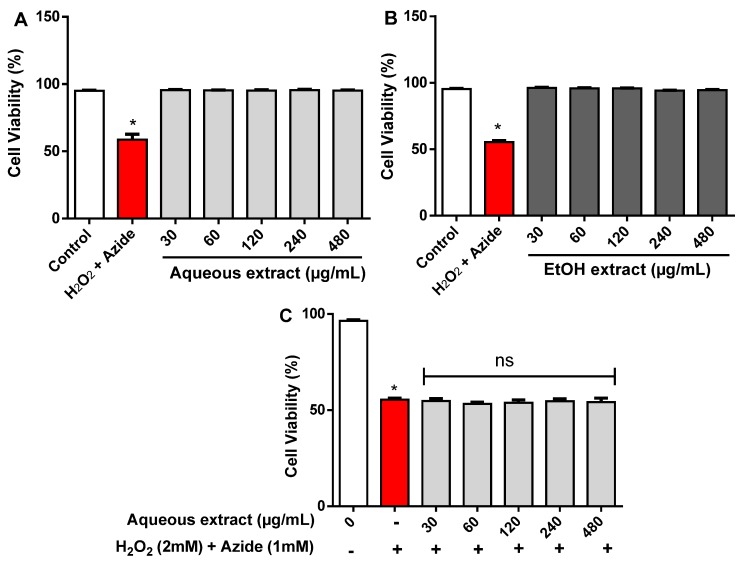

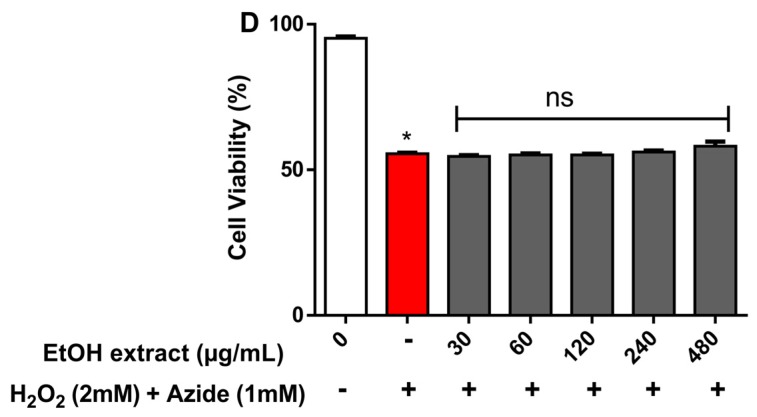
Effect of *R. echinus* leaves extracts on human leukocytes in the absence (**A**,**B**) and presence (**C**,**D**) of H_2_O_2_ (2 mM) + Azide (1 mM). The signs (−) and (+) indicate the absence and presence of the mixture H_2_O_2_ (2 mM) + Azide (1 mM), respectively. Results are expressed as percentage of control. Values are the means of *n* = 3 independent experiments performed in triplicate ± SEM. *: *p* < 0.05 *vs.* control.

The cytotoxicity of *R. echinus* extracts was investigated in human leukocytes using the Trypan blue exclusion method. In this method, dead cells allow passage of trypan blue into the cytoplasm due to loss of membrane selectivity [9,21]. Here, visual analysis of the cells using a Neubauer chamber allowed counting the number of dead (trypan blue positive) and living (trypan blue negative) cells. Exposure for 3 h of human leukocytes to aqueous and ethanolic extracts of *R. echinus* did not modify cell viability, when compared to the control (Figure 6A,B). Cell viability of treated leukocytes was generally greater than 90%, confirming the absence of cytotoxicity of the crude extracts of *R. echinus*. The potential protective effects of both extracts against H_2_O_2_ + azide induced cytotoxicity was also investigated. H_2_O_2_ (2 mM) + azide (1 mM) significantly decreased leukocytes viability when compared to control (*p* < 0.05; Figure 6C,D). However, aqueous (Figure 6C) and ethanolic (Figure 6D) extracts of *R. echinus* did not blunt the cytotoxicity caused by H_2_O_2_ + azide (Figure 6C,D).

### 2.4. Effect of R. echinus Extracts on Osmotic Fragility of Human Erythrocytes

**Figure 7:**
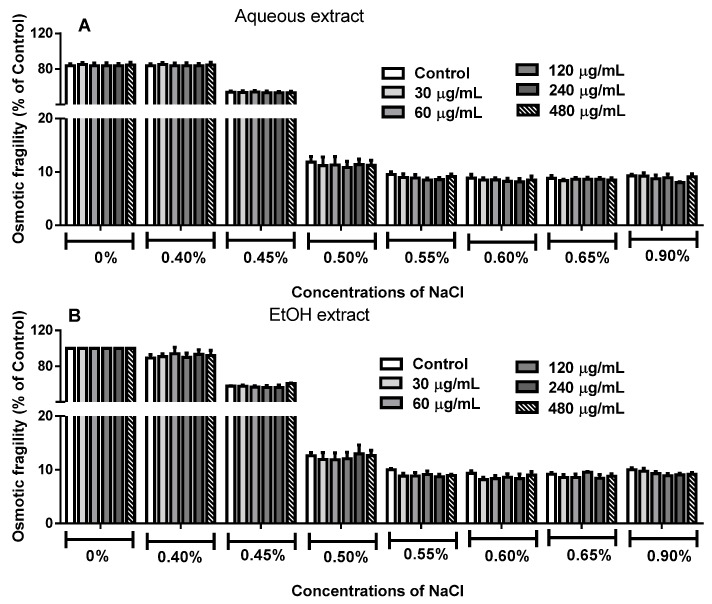
Osmotic fragility of human erythrocytes treated with aqueous (**A**) and ethanolic (**B**) extracts of the leaves of *R. echinus*. Untreated and treated erythrocytes were added to different concentrations of NaCl (0%–0.9%) and incubated for 20 min. The absorbance of the supernatant was measured and the hemolysis in each tube was expressed as percentage of control. Each bar represents the mean of *n* = 3 independent experiments performed in triplicate ± SEM.

The influence of aqueous and EtOH extracts from the leaves of *R. echinus* on human erythrocytes osmotic fragility at different salt concentrations (0%–0.9%) is depicted in Figure 7. The results demonstrated no significant difference between treated and untreated erythrocytes at different salt concentrations in comparison to their respective control (*p* > 0.05; Figure 7A,B).

Osmotic fragility assay is widely used to verify the toxicity of chemicals (and plant extracts) and environmental pollutants on membrane integrity of erythrocytes [9,22,23]. Despite the fact that erythrocytes are well equipped with several biological mechanisms to defend against free radical induced LPO, erythrocyte membrane, however, is prone to oxidative stress, particularly because of the high oxygen tension in the blood and high polyunsaturated fatty acid content [24,25]. Here, the cytotoxic effect of the leaves extracts of *R. echinus* was investigated on human erythrocytes, so as to provide primary information on the interaction between their phytoconstituents and erythrocytes membrane [26,27]. Our results demonstrated that 3 h treatment of human erythrocytes with *R. echinus* extracts did not affect erythrocytes membrane integrity when compared to the control. These results indicate that the chemical constituents of the extracts did not cause gross changes in membrane protein and lipid structure of the cells.

### 2.5. Effect of R. echinus Extracts on DNA Damage

**Figure 8:**
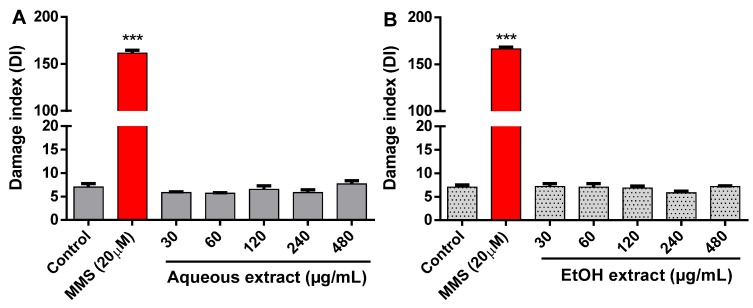
DNA damage of leukocytes treated with the aqueous (**A**) and ethanolic (**B**) leaves extracts of *R. echinus.* Methyl methanesulfonate (MMS) was used as positive control. The results are expressed as means ± SEM of *n* = 3 independent experiments. ***: *p* < 0.001 *vs.* control.

As depicted in Figure 8, methyl methanesulfonate (MMS), used as positive control, caused a dramatic increase in DNA damage when compared with the control (*p* < 0.001). However, no significant difference was found in the DNA damage index (DI) between human leukocytes treated with aqueous (Figure 8A) and EtOH (Figure 8B) extracts, when compared to control (*p* > 0.05).

Comet assay (single-cell gel electrophoresis) is widely used to access DNA damage and repair in individual cells. Studies have indicated that comet assay is a useful tool for the detection of genotoxicity thus, making this assay an important biomarker in toxicological studies [27,28]. In the present study, the potential genotoxic effect of the extracts components at the level of DNA was evaluated in human leukocytes. Our data showed that the leaf extracts of *R. echinus* are not genotoxic. This suggests that the phytoconstituents of these extracts do not/or have not interacted with the DNA. According to Galloway *et al.* [29,30], a situation where DNA damage occurs without a concomitant cytotoxicity is of greater concern. Here, we found no DNA damage and no cytotoxicity. Although it is difficult to extrapolate *in vitro* findings to *in vivo* exposure; nevertheless, we can speculate that the popular use of both extracts may not cause overt toxicity.

## 3. Experimental Section

### 3.1. Chemicals

All chemicals used including solvents were of analytical grade. Methanol, acetic acid, Gallic acid, caffeic acid, ellagic acid and chlorogenic acid were purchased from Merck (Darmstadt, Germany). Gallic, chlorogenic, caffeic, and ellagic acids, quercetin, rutin, and quercitrin, were acquired from Sigma Chemical Co. (St. Louis, MO, USA). 1,1-diphenyl-2-picrylhydrazyl (DPPH), ascorbic acid, malonaldehydebis-(dimethyl acetal) (MDA), thiobarbituric acid (TBA), sodium azide and hydrogen peroxide (H_2_O_2_) were purchased from Sigma Chemical Co.

### 3.2. Plant Material

The leaves *R. echinus* (Nees and Mart) Shauer were collected in Padre Cicero, Crato-Ceará (7°22′S; 39°28′W, 492 m above sea level), Brazil, in January 2014. The plant material was identified by Maria Arlene Pessoa da Silva, and deposited in the Herbarium Caririense Dárdano de Andrade—Lima, Universidade Regional do Cariri (URCA), with the number 7347. To obtain the aqueous extract of *R. echinus,* 300 g of crushed leaves was mixed with 2000 mL of hot water and the mixture was allowed to stand for three days. The mixture was filtered and the filtrate was lyophilized to obtain a dark green solid (3.3% yield). However, for the ethanolic extract, the fresh crushed leaves (300 g) were macerated with 1500 mL of 92% ethanol for three days. On the third day, the suspension was filtered and the filtrate containing the solvent was evaporated under reduced pressure and lyophilized to obtain 1.04% yield of the dry material. The prepared extracts (aqueous and ethanolic) were stored in the freezer until being tested. It should be stressed that the aqueous and ethanolic extracts of *R. echinus* were used in this study on the basis of its popular use in the form of infusion, and also because the roots of the plant are soaked in ethanol prior to use.

### 3.3. Quantification of Compounds by HPLC-DAD

Reverse phase chromatographic analyses were carried out under gradient conditions using C_18_ column (4.6 mm × 150 mm) packed with 5 μm diameter particles; the mobile phase was water containing 1% formic acid (A) and methanol (B), and the composition gradient was: 13% of B until 10 min and changed to obtain 20%, 30%, 50%, 60%, 70%, 20% and 10% B at 20, 30, 40, 50, 60, 70 and 80 min, respectively, following the method described by Pereira *et al.* [31], with slight modifications. Aqueous and ethanolic extracts of *R. echinus* as well as the mobile phase were filtered through 0.45 μm membrane filter (Millipore, Billerica, MA, USA) and then degassed by ultrasonic bath prior to use. The e*x*tracts were analyzed at concentration of 20 mg/mL for the presence or absence of Gallic, chlorogenic, caffeic and ellagic acids, and rutin, quercitrin and quercetin. These compounds were identified by comparing their retention time and UV absorption spectra with those of the commercial standards. The flow rate was 0.7 mL/min, injection volume 40 μL and the wavelength were 257 nm for Gallic acid, 327 nm for chlorogenic, caffeic and ellagic acids, and 365 nm for quercetin, quercitrin and rutin. Stock solutions of standards references were prepared in the HPLC mobile phase at a concentration range of 0.030–0.250 mg/mL for quercetin, quercitrin and rutin; and 0.035–0.300 mg/mL for ellagic, Gallic, caffeic and chlorogenic acids. Chromatography peaks were confirmed by comparing its retention time with those of reference standards and by DAD spectra (200 to 600 nm). All the chromatography operations were carried out at ambient temperature and in triplicates. The limit of detection (LOD) and limit of quantification (LOQ) were calculated based on the standard deviations of the responses and their slopes using three independent analytical curves. LOD and LOQ were calculated as 3.3 and 10 σ/S, respectively, where σ is the standard deviation of the response and S is the slope of the calibration curve.

### 3.4. Determination of Total Phenolics

The determination of total phenolic content was performed by the Folin–Ciocalteu method, as described by Kamdem *et al.* [32], with some modifications [33]. Briefly, 0.5 mL of 2 N Folin–Ciocalteu reagent was added to a 1 mL of ethanolic or aqueous extract of *R. echinus* (0.15 mg/mL) and the mixture was allowed to stand for 5 min before the addition of 2 mL of 20% Na_2_CO_3_. The tubes were then allowed to stand at room temperature for 10 min and the absorbance was measured against water at 730 nm using Shimadzu-UV-1201 (Shimadzu, Kyoto, Japan) spectrophotometer. The phenolic content was expressed in milligram of Gallic acid equivalent per gram of dry extract (mg GAE/g of dry extract). The standard curve obtained with Gallic acid was Y = 29.315 × X − 0.04 (r^2^ = 0.9997).

### 3.5. Antioxidant Activity

#### 3.5.1. DPPH Radical Scavenging Activity

The radical scavenging ability of the aqueous and ethanolic extracts of *R. echinus* was performed using the stable free radical DPPH (1,1-diphenyl-2-picrylhydrazyl) as described by Kamdem *et al.* [32], with some modifications. Briefly, 50 μL of aqueous and ethanolic extracts at different concentrations (30–480 μg/mL) were mixed with 100 μL of freshly prepared DPPH solution (0.3 mM in ethanol). Then, the plate was kept in the dark at room temperature for 30 min. The reduction in the DPPH radical was measured by monitoring the decrease of absorption at 517 nm using a microplate reader (SpectraMax, Sunnyvale, CA, USA). Ascorbic acid was used as standard compound (*i.e.*, positive control). The DPPH radical scavenging capacity was measured using the following equation:

% inhibition = 100 – (A_sample_ − A_blank_)/A_control_ × 100 (1)

where A_sample_ is the absorbance of the tested sample with DPPH; A_blank_, the absorbance of the test tube without adding the DPPH and A_control_, is the absorbance of the DPPH solution.

#### 3.5.2. Production of TBARS from Animal Tissues

The production of thiobarbituric acid reactive substances (TBARS) was measured as described by Ohkawa *et al.* [34], and modified by Barbosa-Filho *et al.* [35]. The rats were killed by decapitation. The whole brain and the liver were quickly removed, placed on ice and weighed. The tissues were immediately homogenized in cold 10 mM Tris-HCl, pH 7.4 (1:10, *w*/*v* for the liver and 1:5, *w*/*v* for brain) and centrifuged at 3600 rpm for 10 min. The pellet was discarded and the supernatant was used to perform the assay. Aliquots of brain or liver homogenates (20 μL) was incubated with 10 μM FeSO_4_ in the presence or absence of extracts (30–480 μg/mL) at 37 °C for 1 h, to induce lipid peroxidation. Subsequently, 40 μL of sodium dodecyl sulphate (8.1%), 100 μL of acetic acid/HCl (pH 3.4) and 100 μL of 0.6% thiobarbituric acid (TBA) were added and incubated at 100 °C for 1 h. After cooling, the samples were centrifuged for 2 min at 6000 rpm and the absorbance of the supernatant was read at 532 nm using an ELISA plate reader (SpectraMax). This work was carried out in accordance with the Guidelines of the Ethical Committee of UFSM and approved by the institutional review board of UFSM (076.2012-2).

#### 3.5.3. Iron Chelating Activity of *R. echinus* Extracts

The chelating capacity of aqueous and ethanolic extracts of the leaves of *R. echinus* was determined according to the modified method of Kamdem *et al.* [27]. The reaction mixture containing 58 μL of saline solution (0.9%, *w*/*v*), 45 μL Tris-HCl (0.1 M, pH, 7.5), 27 μL of extracts (30–120 μg/mL) and 36 μL of 110 μM FeSO4 was incubated for 10 min at 37 °C. Then, 34 μL of 1,10-phenanthroline (0.25%, *w*/*v*) was added and the absorbance of the orange coloured complex formed was measured at 0, 10 and 20 min at 510 nm (against blank solutions of the samples) using microplate reader (SpectraMax). The same procedure was performed for the control (*i.e.*, Fe^2+^), but without the extract. To ascertain the chelating potential of the extracts, we determined the potential reduction of any Fe^3+^ (that might be formed during the incubation periods) by adding the reducing agent, ascorbic acid (to give a final concentration of 5 mM) o the reaction mixture. The absorbance was then determined after 5, 10 and 20 min following ascorbic acid addition. This is because the extracts could be oxidizing Fe^2+^ to Fe^3+^, leading to a decrease in absorbance that was not related to Fe^2+^ chelation. To summarize, the schedule for the evaluation of Fe^2+^ chelation or oxidation by the extracts is presented in Table 1.

### 3.6. Blood Collection and Preparation of Human Leukocytes and Erythrocytes

Heparinized venous blood was obtained from healthy volunteer donors at the hospital of the Federal University of Santa Maria (UFSM), Santa Maria-RS, Brazil (age 26 ± 9). The study was approved by the Ethical Committee of UFSM and registered under the protocol number 0089.0.243.000-07. Human leukocytes and erythrocytes were obtained as previously reported [27,35]. The leukocytes were separated by differential sedimentation rate using 5% dextran and subsequent adjustment of samples to 2 × 10^6^ leukocytes/mL with Hank’s buffered saline solution (HBSS)/heparin (5.4 mM KCl, 0.3 mM Na_2_HPO_4_, 0.4 mM KH_2_PO_4_, 4.2 mM NaHCO_3_, 1.3 mM CaCl_2_, 0.5 mM MgCl_2_, 0.6 mM MgSO_4_, 137 mM NaCl, 10 mM d-glucose, 10 mM Tris-HCl, and heparin 15 IU/mL, adjusted to pH 7.4). However, the erythrocytes were separated by centrifuging the blood sample at 2000 rpm for 5 min at room temperature. The plasma was aspirated and the cell pellet was washed three times with phosphate buffered saline (6.1 mM, pH 7.4, containing 150 mM NaCl).

### 3.7. Determination of Erythrocytes Osmotic Fragility

The influence of the extracts on osmotic fragility of erythrocytes was estimated by measuring their resistance to hemolysis in increasing concentrations of salt solutions as modified by Barbosa-Filho *et al.* [35]. Five hundred microliters of erythrocytes, 100 μL of various concentrations of aqueous and ethanolic extracts (30–480 μg/mL) and 900 μL of phosphate buffer saline (PBS) (6.1 mM, pH 7.4, containing 150 mM NaCl) were pre-incubated for 3 h at 37 °C. After incubation, samples were mixed and centrifuged at 2500 rpm for 10 min and the supernatant was discarded. The erythrocytes were washed twice with PBS, centrifuged at 2500 rpm for 2 min and the supernatant discarded. Treated and untreated erythrocytes (7.5 μL) were then incubated with 1.5 mL of varying concentrations (0%–0.9%) of NaCl, pH 7.4 for 20 min. The samples were homogenized and centrifuged at 2000 rpm for 5 min. The supernatant obtained from each Eppendorf was transferred to microplate and the lysis of erythrocytes was monitored by measuring the absorbance of hemoglobin content in the supernatants at 540 nm using microplate reader (SpectraMax). The results were expressed as percentage of the control.

### 3.8. Genotoxicity by the Comet Assay

The procedure described by Collins [36] was used with some modifications as described by Kamdem *et al.* [27]. Leukocytes were isolated as described earlier and treated with different concentrations of aqueous and ethanolic extracts of *R. echinus* (30–480 μg/mL) for 3 h. The comet assay was performed according to the following steps: (1) 15 μL of leukocyte suspension (2 × 10^6^ leukocytes/mL) was mixed with low-melting agarose; (2) added to 90 mL of 0.75% LMP agarose (*w*/*v*), mixed, and placed on a microscope slide precoated with normal melting point agarose (1% *w*/*v*); (3) a coverslip was added and the samples allowed to solidify at 4 °C; (4) coverslips were removed and slides were placed in a lysis solution (2.5 M NaCl; 100 mM EDTA; 8 mM Tris-HCl; 1% Triton X-100, pH 10–10.5), where they remained for 24 h protected from light; (5) after lysis, the slides were placed in bucket containing neutralizing solution (400 mM Tris-HCl; pH 7.5) for 15 min; (6) then, they were incubated in electrophoretic solution (300 mM NaOH, 1 mM EDTA, pH 13.5) for 20 min at 4 °C under the condition of 25 V, 300 mA, 7W; (7) the slides were washed three times in distilled water and allowed to dry at room temperature; (8) the slides were rehydrated for 3 min in distilled water and fixed for 10 min in 15% trichloroacetic acid, 5% zinc sulfate, and 5% glycerol, then, washed three times in distilled water and allowed to dry at room temperature; (9) the slides were stained with 5% sodium carbonate, 0.1% ammonium nitrate, 0.1% silver nitrate, 0.25% tungstosilicico acid and 0.15% formaldehyde; (10) staining was stopped with 1% acetic acid and air dried sheets; and, finally, (11) slides were visualized and scored according to tail length into five class (from class 0: undamaged, without a tail; to class 4: maximum damage, comet with no heads) under blind conditions by at least two individuals. DNA damage was presented as DNA damage index (DI), which is based on the length of migration. The DI was calculated from cells in different damage classes as follows:

DI = 1 × n1 + 2 × n2 + 3 × n3 + 4 × n4
(2)

where, n1–n4 indicates the number of cells with level 1–4 of damage. Methyl methanesulfonate (MMS) (20 μM) was used as positive control and the negative control with distilled water.

### 3.9. Cytotoxicity by the Trypan Blue Exclusion Method (Cell Viability)

The cell viability was determined using the Trypan blue exclusion method, which assumes that nonviable cells are stained blue [37]. Briefly, 2.5 μL of the extracts (30–480 μg/mL) was added to 497.5 μL of leukocytes suspension and incubated in the presence or absence hydrogen peroxide (2mM) + azide (1 mM), at 37 °C for 3 h. The azide was used to inhibit the activity of catalase in the cell and consequently detect the toxicity induced by H_2_O_2_ used as positive control. Thereafter, 50 μL leukocytes was mixed with 50 μL of 0.4% Trypan blue and allowed to stand for 5 min at room temperature. From the mixture, an aliquot of 10 μL was checked microscopically for viability using a hemocytometer. The viability was as expressed as viable cells in percent of the total cells.

### 3.10. Statistical Analysis

Values were expressed as mean ± standard error of mean (S.E.M). All the analyses were performed using one-way analysis of variance (ANOVA), except for iron chelation, where data were treated with two-way ANOVA followed by Bonferroni post-test. The results were considered significantly different when *p* < 0.05.

## 4. Conclusions

In conclusion, the results presented in this study demonstrated for the first time the *in vitro* antioxidant activity of extracts from the leaves of *R. echinus*, which was evidenced by their potential to scavenge the DPPH radical and inhibit lipid peroxidation in rat brain and liver homogenates. *R. echinus* extracts was neither cytotoxic nor genotoxic to human leukocytes and erythrocytes, respectively, which give indirect support of the popular use of this plant in traditional Brazilian medicine. However, *in vivo* investigation to ascertain the safety of this plant and its extracts needs to be conducted.
